# A longitudinal evaluation of the Center for Epidemiologic Studies-Depression scale (CES-D) in a Rheumatoid Arthritis Population using Rasch Analysis

**DOI:** 10.1186/1477-7525-5-41

**Published:** 2007-07-13

**Authors:** Tanya Covic, Julie F Pallant, Philip G Conaghan, Alan Tennant

**Affiliations:** 1 School of Psychology, University of Western Sydney, Locked Bag 1797, Penrith South DC 1797, NSW, Australia; 2 School of Rural Health, University of Melbourne, 49 Graham Street, Shepparton, 3630, Victoria, Australia; 3 Academic Section of Musculoskeletal Disease, Leeds Institute of Molecular Medicine, Faculty of Medicine and Health, University of Leeds, 36 Clarendon Road, Leeds, LS2 9NZ, UK

## Abstract

**Background:**

The aim of this study was to test the internal validity of the total Center for Epidemiologic Studies-Depression (CES-D) scale using Rasch analysis in a rheumatoid arthritis (RA) population.

**Methods:**

CES-D was administered to 157 patients with RA over three time points within a 12 month period. Rasch analysis was applied using RUMM2020 software to assess the overall fit of the model, the response scale used, individual item fit, differential item functioning (DIF) and person separation.

**Results:**

Pooled data across three time points was shown to fit the Rasch model with removal of seven items from the original 20-item CES-D scale. It was necessary to rescore the response format from four to three categories in order to improve the scale's fit. Two items demonstrated some DIF for age and gender but were retained within the 13-item CES-D scale. A new cut point for depression score of 9 was found to correspond to the original cut point score of 16 in the full CES-D scale.

**Conclusion:**

This Rasch analysis of the CES-D in a longstanding RA cohort resulted in the construction of a modified 13-item scale with good internal validity. Further validation of the modified scale is recommended particularly in relation to the new cut point for depression.

## Background

Rheumatoid arthritis (RA) is one of the most common chronic inflammatory joint diseases [1] and is associated with depression [2]. The reported prevalence of depression in this population ranges from 13 to 20% [3] when based on psychiatric assessment, but may be as high as 40% when based on self-reported assessment [4]. Indeed, in a UK study of over 7000 patients with RA, 19% were identified as clinically depressed at some point during the disease course [5] clearly indicating that the co-morbidity of depression in RA significantly exceeds the rates of depression in a general community (2–4%) or primary care (5–10%) population [6].

Depression in RA is closely associated with pain, work disability, health services utilisation, poor adherence to treatment and even suicide (see Sheehy [2] for review) making the identification and treatment of depression in RA paramount to the overall management of RA. It has been suggested that improving the awareness of depression in RA could be achieved with regular mood assessment by rheumatologists and/or clinical nurse specialists [2]. The use of self-report scales, while not substituting for a psychiatric clinical assessment, may be useful as screening tools to identify patients with RA who may be at risk of depression, and to use as an outcome measure.

The Center for Epidemiologic Studies-Depression (CES-D) scale is one of the commonly used depression measurement tools originally developed for use in the general population [7]. It has also been found to be valid and reliable in identification of individuals at high risk of developing major depression in clinical populations including RA [8], brain injury [9], multiple sclerosis [10], cancer [11] and stroke [12].

Although there is strong psychometric support for the CES-D its structural validity has been questioned [13]. The CES-D was developed based on Beck's [14]cognitive model of depression representing four factors, namely negative affect (e.g. item 14 '*I felt lonely*'), positive affect (e.g. item 16 '*I enjoyed life*'), interpersonal difficulties (e.g. item 15 '*People were unfriendly*') and somaticism (e.g. item 11 '*My sleep was restless*'). While a number of studies have replicated the original factors, those findings could not be generalised to an RA population as there has been evidence of criterion contamination with some somatic items being disease related (e.g. item 7 '*I felt that everything I did was an effort*') rather than indicative of depression [15,16]. Rhee et al [8] in a longitudinal examination of CES-D in a sample of 685 patients with RA found support for the original four factors but also evidence of criterion contamination in this population. It has been suggested that the four theory-driven factors of the CES-D are interrelated in the single-factor hierarchical model [17] and that the use of factor analytic methods may mask a general psychological distress factor [18].

Exploratory and confirmatory factor analyses have been commonly used to test the CES-D latent structure, however these techniques are sample dependent [8], and tend to produce different findings [13]. They also fail to "identify dimensions on which the summed total score is a meaningful and sufficient statistic" [19] whereby items are equivalent in meaning across individuals [20]. Consequently there is a growing use of modern psychometric techniques such as Rasch analysis [21] and the related models within Item Response Theory [22]. These approaches are increasingly being used in the development of new scales and in the improvement of existing scales that measure latent traits such as depression, by establishing their fundamental measurement ability [23]. Thus, Rasch analysis can provide true interval scaling, significant information about the respondents with extreme scores, and a more comprehensive understanding of the underlying latent structure [13,23]. As such, Rasch analysis has the potential to improve existing scales perhaps with fewer and more relevant questions without compromising the screening efficacy of scales such as those that assess psychological distress [24].

Only a few studies, however, have used Rasch models to test the CES-D and these include a test of population differences (stroke vs primary-care patients) [12]; CES-D mode effect (phone vs mail interview) in a depressed population [25]; and the development of a short-form CES-D in a general population [13]. No studies have been conducted to test the CES-D in a RA population or to test the scale's stability over time which is an important indicator of the scale's validity and its utility as an outcome measure.

### Current study

The aim of this study, therefore, was to use Rasch analysis to test the CES-D's internal validity in terms of unidimensionality and the stability of responses across time (three time points over a period of 12 months), age (three groups: ≤53 years old; 54–65 years old; 66+ years old) and gender (male/female) in an RA population. The sequence of Rasch analysis is briefly explained below, while a more detailed introduction may be found elsewhere [26].

## Methods

### Rasch Analysis

The Rasch model is a unidimensional model based on the basic probability model that expects the only relationship between a respondent and the response to the scale item to be the respondent's ability on a given latent trait. That is, the probability *p *that a person *n *will affirm an item *i *is a logistic function of the difference between the person's ability *θ *and the difficulty of the item *b*, and only a function of that difference:

pni=e(θn−bi)1+e(θn−bi)
 MathType@MTEF@5@5@+=feaafiart1ev1aaatCvAUfKttLearuWrP9MDH5MBPbIqV92AaeXatLxBI9gBaebbnrfifHhDYfgasaacH8akY=wiFfYdH8Gipec8Eeeu0xXdbba9frFj0=OqFfea0dXdd9vqai=hGuQ8kuc9pgc9s8qqaq=dirpe0xb9q8qiLsFr0=vr0=vr0dc8meaabaqaciaacaGaaeqabaqabeGadaaakeaacqWGWbaCdaWgaaWcbaGaemOBa4MaemyAaKgabeaakiabg2da9maalaaabaGaemyzau2aaWbaaSqabeaacqqGOaakiiGacqWF4oqCcqWGUbGBcqGHsislcqWGIbGydaWgaaadbaGaemyAaKgabeaaliabcMcaPaaaaOqaaiabigdaXiabgUcaRiabdwgaLnaaCaaaleqabaGaeiikaGIae8hUdeNaemOBa4MaeyOeI0IaemOyai2aaSbaaWqaaiabdMgaPbqabaWccqGGPaqkaaaaaaaa@4832@

where *p*_*ni *_is the probability that person *n *will affirm the item, whereby *θ *is the person's level of depression, and *b *is the level of depression expressed by a positive response to the item. The formulae can be expressed as a logit model:

ln⁡(Pni1−Pni)=θn−bi
 MathType@MTEF@5@5@+=feaafiart1ev1aaatCvAUfKttLearuWrP9MDH5MBPbIqV92AaeXatLxBI9gBaebbnrfifHhDYfgasaacH8akY=wiFfYdH8Gipec8Eeeu0xXdbba9frFj0=OqFfea0dXdd9vqai=hGuQ8kuc9pgc9s8qqaq=dirpe0xb9q8qiLsFr0=vr0=vr0dc8meaabaqaciaacaGaaeqabaqabeGadaaakeaacyGGSbaBcqGGUbGBdaqadaqaamaalaaabaGaemiuaa1aaSbaaSqaaiabd6gaUjabdMgaPbqabaaakeaacqaIXaqmcqGHsislcqWGqbaudaWgaaWcbaGaemOBa4MaemyAaKgabeaaaaaakiaawIcacaGLPaaacqGH9aqpiiGacqWF4oqCdaWgaaWcbaGaemOBa4gabeaakiabgkHiTiabdkgaInaaBaaaleaacqWGPbqAaeqaaaaa@4345@

where *ln *is the normal log, *P *is the probability of person *n *affirming item *i*; *θ *is the person's level of depression, and *b *is the level of depression expressed by the item. Both item and person parameter estimates are on the same log-odds units (logit) scale, allowing for a linear transformation of the raw score.

When the model is applied to the polytomous case (as distinct from measures that have a dichotomous response option), it is referred to as the rating scale model [27]. A further development of this model allows thresholds (0.5 probability point between adjacent categories) to vary in distance across items, and is known as the partial credit model [28], as expressed below:

ln⁡(Pnij1−Pnij−1)=θn−bij⥄
 MathType@MTEF@5@5@+=feaafiart1ev1aaatCvAUfKttLearuWrP9MDH5MBPbIqV92AaeXatLxBI9gBamXvP5wqSXMqHnxAJn0BKvguHDwzZbqegyvzYrwyUfgarqqtubsr4rNCHbGeaGqiA8vkIkVAFgIELiFeLkFeLk=iY=Hhbbf9v8qqaqFr0xc9pk0xbba9q8WqFfeaY=biLkVcLq=JHqVepeea0=as0db9vqpepesP0xe9Fve9Fve9GapdbaqaaeGacaGaaiaabeqaamqadiabaaGcbaGagiiBaWMaeiOBa42aaeWaaeaadaWcaaqaaiabdcfaqnaaBaaaleaacqWGUbGBcqWGPbqAcqWGQbGAaeqaaaGcbaGaeGymaeJaeyOeI0Iaemiuaa1aaSbaaSqaaiabd6gaUjabdMgaPjabdQgaQjabgkHiTiabigdaXaqabaaaaaGccaGLOaGaayzkaaGaeyypa0dcciGae8hUde3aaSbaaSqaaiabd6gaUbqabaGccqGHsislcqWGIbGydaWgaaWcbaGaemyAaKMaeeOAaOgabeaakiaaykW6aaa@5B07@

In this study, the test of fit of the Rasch model was conducted by use of the RUMM2020 program [29]. Fit is assessed using two statistics, namely residuals and chi-square probability values. Residuals values greater than +/-2.50 and/or the chi-square probability values <0.05 are indicative of item misfit. High positive fit residual values suggest low levels of discrimination and poor fit to the model, whereas high negative fit residual values may be indicative of item dependency or redundancy.

As well as testing the fit of the data to model expectations, Rasch analysis allows an evaluation of the scoring structure of items, that is, do the response categories work as intended? This is indicated by ordered thresholds. The term *threshold *refers to the point between two response categories where either response is equally probable. It is expected that individuals with lower levels of the trait, in this instance depression, would endorse low scoring responses, while respondents with high levels of the trait would endorse high scoring responses, resulting in ordered thresholds. In addition, an examination of the lack of invariance by group is undertaken and referred to as Differential Item Functioning (DIF). In the current study this is investigated for time point (Time 1, Time 2, Time 3), as well as for gender (male/female) and age (three groups: ≤53 years old; 54–65 years old; 66+ years old). This type of analysis investigates whether or not the structure of the scale stays the same across groups, a requirement for valid group comparisons. Thus, to be able to compare patients across time, the scale must be stable, else observed differences may be confounded by the fact that, for example, a raw score of 25 at time 1 does not mean the same as a raw score of 25 at time 2. Both chi-square fit, and the ANOVA DIF tests have significance levels set at 0.05, Bonferroni adjusted for the number of tests being undertaken at any stage.

Finally, when satisfied with fit to the model, threshold ordering and absence of DIF, a formal test of unidimensionality is undertaken by a Principal Components Analysis (PCA) of the residuals. The absence of any meaningful pattern in the residuals will be deemed to support the assumption of unidimensionality of the scale [30]. This is formally tested by allowing the correlation between items and the first residual factor to determine 'subsets' of items and then testing, using a series of independent t-tests, to see if a person's estimate derived from each subset significantly differ [30]. For a unidimensional solution it would be expected that, given the difference in estimates are normally distributed, no more than five percent of such tests would be outside the range ± 1.96. For values falling outside this recommended range, a 95% confidence interval for the binomial test of proportions of the observed value is applied, and if the expected value of five percent falls within the confidence interval then the scale is deemed to be unidimensional.

### Measures

CES-D is a 20-item scale designed to measure depressive symptoms experienced in the past week [7]. Responses range from 0 to 3: 0 = *Rarely or none of the time (less than 1 day)*; 1 = *Some or a little of the time (1–2 days)*; 2 = *Occasionally or a moderate amount of the time (3–4 days)*; and 3 = *Most or all of the time (5–7 days)*. Four of the items (items 4, 8, 12 & 16) are positively worded and therefore should be reverse-scored. The CES-D total score is calculated by adding the scores for all 20 items giving a range from 0 to 60, with the suggested cut-off of 16 as indicative of probable clinical depression. In the RA population it has been suggested that a cut-off of 19 may be more appropriate because of the problem of criterion contamination with somatic items [31].

### Study Participants

Raw scores for the CES-D items were obtained from a dataset with 157 RA participants who completed a range of psychological assessments across three time points within a 12-month period. The aim of the original study was to monitor depression over time in relation to clinical and other psychological outcomes. The retention rate at the second and third data collection points (Time 2 and Time 3) was 85% and 83% respectively. The mean age of participants was 57.85 (SD = 12.24) and 76% were female. RA duration ranged from six months to 47 years, with a mean of 13.07 (SD = 9.45) years. CES-D depression scores across the three measurement points were Time 1: M = 15.94 (SD = 11.92), Time 2: M = 14.30 (SD = 12.14), and Time 3: M = 14.42 (SD = 11.81). Further details of the participants and other assessments are reported elsewhere [4,32].

Informed consent was obtained from all participants and the study was approved by the relevant ethics committee. The participants were recruited through three private rheumatology clinics and had confirmed clinical diagnosis of RA [33] and were currently medically managed for their condition.

## Results

### Overall fit of the CES-D scale

Of the 157 participants at Time 1, 134 at Time 2 and 131 at Time 3, 395 were usable for Rasch analyses. Initial inspection of the scale showed poor overall fit to the Rasch model as evident in the standardised item Fit Residual statistic (mean = 0.039, SD = 3.14) and the item trait-interaction statistic (*χ*^2 ^= 577.79, df = 160, p < 0.001).

The pattern of item response thresholds was then examined, cross-sectionally (at each of the three time points) and longitudinally (three times points merged) to assess if disordering of thresholds may be contributing to misfit to the model. In the initial inspection, the thresholds of items 4, 9, 15 and 19 were found to be disordered (see Table 1 for item wording). The disordering of the thresholds suggests that respondents were not able to reliably distinguish the middle response categories ('*some or a little of the time*' and '*occasionally or a moderate amount of the time*'). Initially rescoring of just those four items did not improve fit to the model. It was decided to rescore all items by merging the two middle categories, thus reducing the number of response categories from four to three, and changing the scoring from 0123 to 0112. Although this represents a change to the original format of the CES-D, it more closely represents the actual response patterns of the respondents in this sample.

**Table 1:** Final fit of the CES-D items to the Rasch model

**CESD Item**	**CESD Item Name**	**Location**	**SE**	**Fit res.**	**DF**	**ChiSq.**	**DF**	**Prob.**
1	Bothered	-0.184	0.119	-0.163	328.47	4.792	6	0.570797
3	Blues	0.311	0.118	-2.449	330.31	10.196	6	0.116651
5	Concentrate	-0.109	0.115	-0.336	330.31	5.601	6	0.469291
6	Depressed	-0.643	0.111	-2.608	326.64	8.055	6	0.23407
7	Effort	-1.371	0.111	0.959	330.31	7.324	6	0.291895
9	Failure	0.58	0.12	-0.487	330.31	1.726	6	0.943111
10	Tearful	-0.346	0.109	-2.755	329.39	10.839	6	0.093481
13	Talked less	-0.003	0.115	0.284	329.39	16.091	6	0.013276
14	Lonely	-0.507	0.105	-1.343	328.47	3.495	6	0.744636
15	Others unfriendly	1.574	0.151	-1.471	330.31	6.255	6	0.395291
17	Crying spells	0.142	0.112	-0.962	329.39	6.047	6	0.417915
19	Others dislike	1.177	0.137	1.6	330.31	13.248	6	0.039264
20	Not get going	-0.62	0.117	-0.589	329.39	3.695	6	0.717908

Next, items were examined individually for fit to the Rasch model. A number of misfitting items were identified. Items were selected for removal if they recorded significant chi-square probability values or high positive or high negative residual values. Items were removed one at the time, with the overall model fit and individual item statistics checked after each step, until a satisfactory model was achieved as indicated by a non-significant chi-square value. In the final solution a total of seven items were removed: items 4, 11, 8, 16, 12, 18 and 2 (listed in order of their removal). The final individual item Fit Residual mean was -0.794 (SD = 1.335); the person Fit Residual mean was -0.432 (SD = 1.150) and the total chi-square interaction value was 97.364 (df = 78, p = 0.068), all of which indicated fit to the Rasch model. The final individual fit statistics are provided in Table 1. The person separation reliability, which is equivalent to Cronbach's alpha, for the final 13-item solution was found to be very good (0.906).

**Figure 1:**
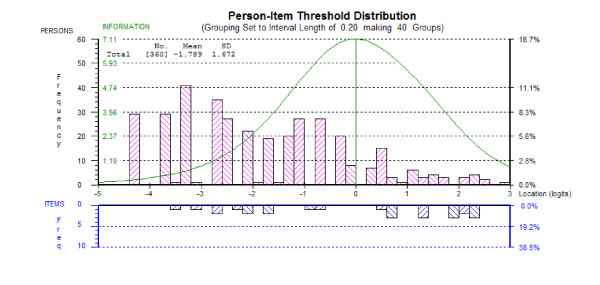
Person item distribution graph for 13-Item CES-D scale.

Figure 1 shows the targeting of the revised scale in this sample, with the distribution of persons shown in the top half and the item thresholds in the bottom half. The overall mean person logit is -1.784, which shows that this group of patients had levels of depression somewhat lower than the target for the scale (which would be indicated by a mean of zero).

### Differential Item Functioning

**Figure 2:**
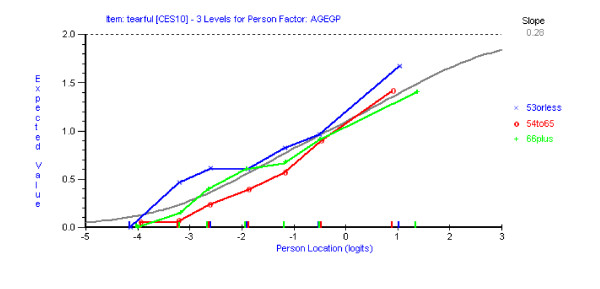
Item characteristic curve for item 10 DIF for age groups.

**Figure 3:**
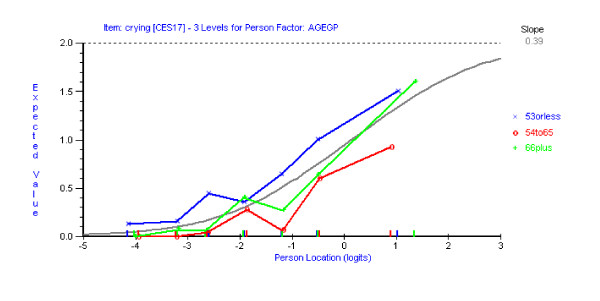
Item characteristic curve for item 17 DIF for age groups.

**Figure 4:**
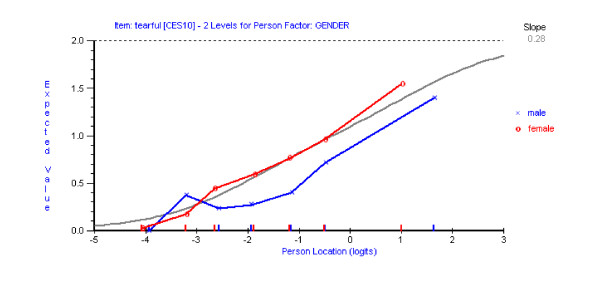
Item characteristic curve for item 10 DIF for gender.

**Figure 5:**
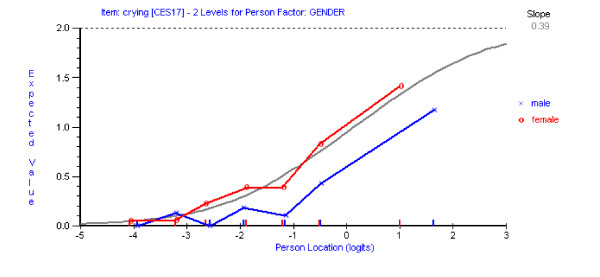
Item characteristic curve for item 17 DIF for gender.

Having reached a satisfactory fit to the Rasch model with the remaining 13 items of the CES-D scale, assessment of differential item functioning (DIF) was conducted using both statistical and graphical procedures. Graphs were plotted to compare item location with respect to time (three time points), age (three age groups) and gender (male/female). No DIF for time point was detected for any item. Using a Bonferroni-adjusted p value item 10 ('*I felt tearful*') and 17 ('*I had crying spells*') showed significant uniform DIF for both age (Figures 2 &3) and gender (Figures 4 &5). Tukey post-hoc tests showed that the participants aged 53 yrs or less, were significantly more likely to endorse those items than the other two age groups (54 yrs to 65 yrs and 66 plus yrs). In terms of the gender differences females were more likely to endorse items 10 and 17 than males.

### Dimensionality

To test the unidimensionality of the 13-item CES-D scale, a principal component analysis was conducted on the residuals in order to identify the two most divergent subsets of items as indicated by positive loadings (Set 1) and negative loadings (Set 2) on the first factor (Table 2). The person ability estimates based on the subsets of items were then generated and compared using a series of independent t-tests (one for each case)[30]. It was found that 23 (6.39%) of the 360 t-tests showed significant differences in the estimates generated, which is non-significant when 95% confidence intervals from a Binomial distribution are applied to this proportion. This supports the unidimensionality of the 13-item CES-D scale.

**Table 2:** Principal component analysis of the residuals showing loadings on the first component extracted

**Item**	**Loading**
CES-D3	**-0.411**
CES-D5	**-0.406**
CES-D1	**-0.399**
CES-D13	**-0.218**
CES-D9	**-0.198**
CES-D7	**-0.102**
CES-D20	**-0.067**

CES-D6	0.012
CES-D14	0.110
CES-D15	0.115
CES-D19	0.239
CES-D10	0.670
CES-D17	0.763

### Cut Point

**Figure 6:**
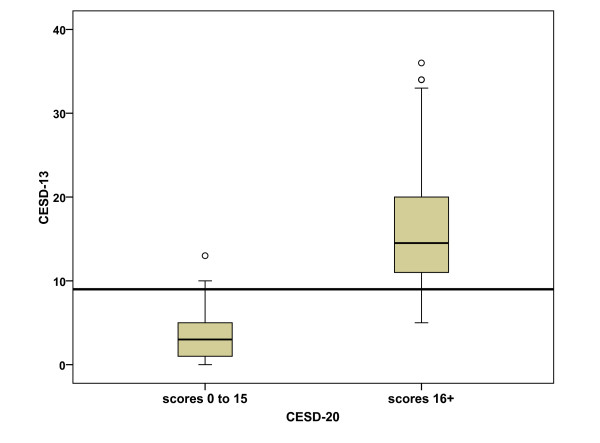
Boxplot comparing cut point of 9 on CES-D 13 with the original cut point of 16 on CES-D 20. The recommended cut point of 9 is shown as a horizontal line.

In order to determine the cut point for depression in the 13-item scale, the logit equivalent of the cut point of 16 in the full CES-D scale was calculated and then applied to the 13-item CES-D scale. The cut point of 16 out of 60 equates to a logit score of -0.89 which when applied to the 13-item scale (with 4 categories) equates to a cut-off point of 9. Of the 41% of cases identified as depressed using the original CESD-20, 96.3% were so classified using the CESD-13, indicating excellent agreement (kappa = 0.86). The very high sensitivity (89.6%) and specificity (95.8%) values also support the use of the cut point value of 9 on the CESD-13. Figure 6 shows a boxplot with the CESD-13 scores plotted separately for the individuals with scores above and below the cutpoint of 16 on the CESD-20.

## Discussion

The aim of the current study was to use Rasch analysis to test the psychometric properties of CES-D in the RA population, and its response stability across time, age and gender. Seven items were found to misfit the scale and were subsequently removed. Four of these items were positively worded (item 4, *feeling as good as others*; item 8, *feeling hopeful*; item 12, *feeling happy*; & item 16, *enjoying life*), while the other removed items included one from the original CES-D 'depressed affect' factor (item 18, *feeling sad*) and two from the somatic factor (item 2, *poor appetite *& item 11, *restless sleep*). The four positive items comprise the CES-D's factor 'positive affect', however other studies have suggested that the wording and the response pattern in scales may produce an artifactual factor structure [34] and as such have a significant impact on the psychometrics of scales [35]. A study of cancer patients (n = 475) and healthy general population (n = 255) using the CES-D suggested that the negative worded items (16 items) and the positive worded items (four items) may measure different constructs and the authors recommend that only negative items should be used to measure depression [36].

The collapsing of response categories in the current study did not significantly improve fit to model expectations. While the collapsing pattern made sense from a distributional point of view, further work needs to be done in larger samples to see if the current strategy is optimal. Two items, item 10 (*tearful*) and item 17 (*crying spells*) were also found to display DIF across age and gender, with younger participants and females more likely to endorse them than older and male participants. These age and gender differences may make them potentially unsuitable for inclusion in core sets of scale items, but they may be clinically informative [12]. Again, replication of these results would strengthen the case for retaining, or excluding these items on the basis of DIF.

The results of this study differ from two other studies that used Rasch modelling with the CES-D. The Cole et al [13] study aimed to develop a short-form CES-D with the selection of items partly driven by the preservation of the four-factors identified in the full CES-D scale. As such, their 10-item short CES-D contains two of the positive items rejected in our study (item 4, *feeling as good as others *& item 8, *feeling hopeful about the future*) but shares the other four removed items (items 2, 11, 16 & 18) in our study. In addition, item 17, which was retained in our study (although indicating some differential item functioning for age and gender), was removed in the Cole et al' [13] study. The Pickard et al [12] study compared depressed stroke patients (n = 32) and depressed primary-care patients (n = 366) and found that while the 20-item CES-D scale had a satisfactory fit in the primary-care group, items 11, 17, 15 and 4 were misfitting in the stroke group. Furthermore, when the two groups were compared, items 2, 11, 17 and 19 demonstrated significant DIF. While there is some overlap in the findings across these three studies, the differences may be due to the study focus (i.e. short scale version), methodology or populations and further exploration of CES-D is warranted using the Rasch model before recommending an altered/reduced version of the scale for clinical application.

In terms of the scale's targeting, the results of this study indicate a floor effect with the clustering of participants at the low end of the scale (indicating low levels of depression). Furthermore, the distribution of item thresholds indicate a shortfall in their distribution across the middle of the construct (Figure 1) suggesting the potential for adding items which reflect levels of depression at the middle of the scale. However, the function of CES-D is to identify participants who are at risk/likely to be clinically depressed. As such the sensitivity of the scale at the cut point is of primary consideration. By equating the two scales (the CES-D 20 and CES-D 13) it was determined that the cut point in the shorter scale is consistent with the cut point [16] used with the original scale, showing excellent specificity and sensitivity against the original.

The current study is limited in terms of the relatively small sample and our findings should be further tested in other RA populations, for example, those with predominantly new onset disease. Furthermore, the modified scale and the proposed cut point for depression requires confirmation against other validated measurements (i.e. clinical assessment such as disease duration, disease activity and pain levels as well as other depression scales and psychological outcomes measurements). The strengths of this study, however, are in the use of a modern, sophisticated statistical approach, Rasch modelling, to test the psychometric properties of the scale; and the use of longitudinal data to test the stability of the CES-D across time.

Notwithstanding the study limitations, our findings raise doubts about the internal construct validity of the full 20-item scale for those with RA, and suggest that the identification of clinical depression may be compromised by the scale's multidimensionality.

## Conclusion

In conclusion, the revised CES-D scale shows promising internal validity for RA when evaluated under the strict requirements of the Rasch measurement model. We recommend further validation studies of this revised scale against a clinical assessment and other depression scales in RA. Further testing in other clinical populations is needed to resolve the issues of category ordering and DIF.

## Competing interests

The author(s) declare that they have no competing interests.

## Authors' contributions

All authors contributed to the current study's conceptualization, statistical analyses, interpretation, and preparation of the article and approved the final manuscript.
